# Incidental Use of Beta-Blockers Is Associated with Outcome of Metastatic Colorectal Cancer Patients Treated with Bevacizumab-Based Therapy: A Single-Institution Retrospective Analysis of 514 Patients

**DOI:** 10.3390/cancers11121856

**Published:** 2019-11-25

**Authors:** Ondrej Fiala, Pavel Ostasov, Ondrej Sorejs, Vaclav Liska, Tomas Buchler, Alexandr Poprach, Jindrich Finek

**Affiliations:** 1 Department of Oncology and Radiotherapy, Medical School and University Hospital in Pilsen, Charles University, alej Svobody 80, 30460 Pilsen, Czech Republic; sorejso@fnplzen.cz (O.S.); finek@fnplzen.cz (J.F.); 2 Biomedical Center, Faculty of Medicine in Pilsen, Charles University, alej Svobody 76, 32300 Pilsen, Czech Republic; pavel.ostasov@lfp.cuni.cz (P.O.); liskav@fnplzen.cz (V.L.); 3 Department of Surgery, Medical School and University Hospital in Pilsen, Charles University, alej Svobody 80, 30460 Pilsen, Czech Republic; 4 Department of Oncology, First Faculty of Medicine, Charles University and Thomayer Hospital, Videnska 800, 14059 Prague, Czech Republic; tomas.buchler@ftn.cz; 5 Department of Comprehensive Cancer Care and Faculty of Medicine, Masaryk Memorial Cancer Institute and Masaryk University, Zluty kopec 7, 65653 Brno, Czech Republic; poprach@mou.cz

**Keywords:** colorectal cancer, bevacizumab, beta-blocker, outcome, hypertension

## Abstract

*Background:* Beta-adrenergic signalling plays an important role in several cancer-related processes, including angiogenesis. The impact of beta-blocker use on prognosis of cancer patients treated with antiangiogenic agents is unclear. The aim of this study was to evaluate the association between the incidental use of beta-blockers and the outcomes of patients with metastatic colorectal cancer (mCRC) treated with bevacizumab-based therapy. *Methods:* Clinical data from 514 mCRC patients treated with bevacizumab between 2005 and 2019 were analysed retrospectively. The association of progression-free survival (PFS) and overall survival (OS) with the incidental use of beta-blockers and other common antihypertensive drugs was assessed. *Results:* The median PFS and OS for patients using beta-blockers was 11.40 (95% confidence interval (CI) 10.10–13.61) months and 26.8 (95% CI 22.2–32.2) months compared with 8.30 (95% CI 7.80–9.57) and 21.0 (95% CI 17.8–23.8) months for patients not using beta-blockers (*p =* 0.006 and *p =* 0.009, respectively). In the Cox multivariate analysis, the use of beta-blockers was a significant factor predicting both PFS (hazard ratio (HR) = 0.763 (95% CI 0.606–0.960), *p =* 0.021) and OS (HR = 0.730 (95% CI 0.560–0.951), *p =* 0.020). *Conclusions:* The results of the present retrospective study suggest that there is a significant association between the use of beta-blockers and favourable outcomes of mCRC patients treated with bevacizumab-based therapy.

## 1. Background

Colorectal cancer (CRC) is one of the most common malignancies in developed countries [1]. Bevacizumab represents one of the established targeted agents for the treatment of metastatic CRC (mCRC). A recombinant humanised monoclonal antibody directed against vascular endothelial growth factor A (VEGF-A), it is commonly used in combination with standard chemotherapy regimens consisting of 5-fluorouracil and oxaliplatin and/or irinotecan. The efficacy and safety of bevacizumab in the treatment of patients with mCRC have been demonstrated in randomised phase III clinical trials as well as observational studies [2,3,4,5,6].

Beta-adrenergic receptor antagonists (beta-blockers) have been widely used for the treatment of arterial hypertension and ischaemic heart disease for decades. There is a growing body of evidence that beta-adrenergic receptor downstream signalling plays an important role in several cancer-related processes, such as cell proliferation, apoptosis, invasion and angiogenesis [7]. Experimental studies have suggested the antitumour activity of beta-blockers, but the impact of beta-blocker use on outcomes in cancer patients is still poorly understood [8,9,10,11,12,13,14]. Although several retrospective studies have shown that the use of beta-blockers is associated with better prognosis of patients with various cancers, there are only limited data on the role of beta-blockers in patients with mCRC, particularly in those treated with targeted agents. The aim of this study was to evaluate the association between the incidental use of beta-blockers and the outcomes of patients with metastatic colorectal cancer treated with a combination of bevacizumab and chemotherapy.

## 2. Materials and Methods

### 2.1. Study Design

Clinical data of patients with mCRC treated with a combination of bevacizumab and chemotherapy were analysed retrospectively. The association of progression-free survival (PFS) and overall survival (OS) with the incidental use of beta-blockers as well as other commonly used antihypertensive drugs, including angiotensin-converting-enzyme inhibitors (ACE inhibitors), angiotensin II receptor blockers (sartans) and calcium channel blockers, was assessed. The data on clinical characteristics, including the use of antihypertensive drugs and the follow-up, were obtained from medical records.

### 2.2. Patients and Treatment

In total, 514 adult patients with histologically confirmed mCRC treated with bevacizumab-based therapy between 2005 and 2019 at the Department of Oncology and Radiotherapy, Medical School and University Hospital Pilsen, Czech Republic were included in the study. Baseline characteristics of the patient group are described in Table 1.

Bevacizumab (Avastin, F. Hoffman-La Roche Ltd., Basel, Switzerland) was administered in standard approved doses (5.0 mg/kg every 14 d or 7.5 mg/kg every 21 d) in combination with chemotherapy or as a single agent in the maintenance setting. The chemotherapy regimens included 5-fluorouracil and leucovorin in combination with oxaliplatin (FOLFOX) or irinotecan (FOLFIRI), or 5-fluorouracil and leucovorin (FUFA); capecitabine in combination with oxaliplatin (XELOX) or irinotecan (XELIRI), or capecitabine alone; oxaliplatin alone; and irinotecan alone. None of the patients had received prior antiangiogenic therapy. The status of the antihypertensive therapy was assessed at the start of the treatment with bevacizumab-based therapy and all the antihypertensive drugs were administered orally at individual doses. Bevacizumab was administered in patients with compensated arterial hypertension.

The protocol of the study and the informed consent form for participants were approved by the Ethical Committee of the Medical School and University Hospital in Pilsen on 13 June 2016 and complied with the International Ethical Guidelines for Biomedical Research, the Declaration of Helsinki and local laws. Informed consent with subsequent analysis of the follow-up data was obtained from all the participants.

### 2.3. Outcome Assessment

The clinical status of the patients was assessed continuously during visits at prespecified time points. Physical examination and routine laboratory tests were performed every 2 weeks, and computed tomography (CT) was performed every 3–4 months during the treatment. The objective tumour response was assessed locally by the attending physician using Response Evaluation Criteria in Solid Tumors (RECIST) version 1.1 [15].

### 2.4. Statistical Analysis

Descriptive statistics and standard frequency tables were used to characterise the sample dataset. PFS was determined from the date of treatment initiation until the date of first documented progression or death. OS was determined from the date of treatment initiation until the date of death. In both cases, patients without event were censored at the date of last known follow-up. PFS and OS were estimated using the Kaplan–Meier method and all point estimates were accompanied by two-sided 95% confidence intervals. The statistical analysis was performed using R (version 3.5.2) [16] with packages survival (version 2.43) [17] and survminer (version 0.4.3) [18] for survival analysis and visualisation. The log-rank test was used for assessment of statistical significance of the differences in survival according to treatment. A multivariate Cox proportional hazards model was used to evaluate the effect of all potential prognostic factors on the survival indicators and a univariate Cox model was used to determine the effect of individual antihypertension treatments on the survival indicators. The Wald test was used for assessment of statistical significance of hazard ratios. The level of statistical significance was set at α = 0.05 and all reported *p*-values are two-tailed.

## 3. Results

### 3.1. Patient Characteristics

The study included 514 patients. The baseline patient characteristics are summarised in Table 1. At the time of bevacizumab initiation, 126 (24.5%) patients were using beta-blockers, 163 (31.7%) were using ACE inhibitors, 48 (9.3%) were using sartans and 102 (19.8%) were using calcium channel blockers. Patients using more than one antihypertensive drug were not excluded. At the time of data analysis, 440 (85.60%) patients progressed and 345 (67.12%) patients died. The median follow-up time was 519 d.

Median PFS and OS for the whole cohort were 9.18 months (95% confidence interval (CI) 8.3–10.1) and 22.3 months (95% CI 20.6–24.5), respectively.

### 3.2. Outcome of Patients According to the Use of Beta-Blockers and Other Antihypertensive Drugs

**Figure 1:**
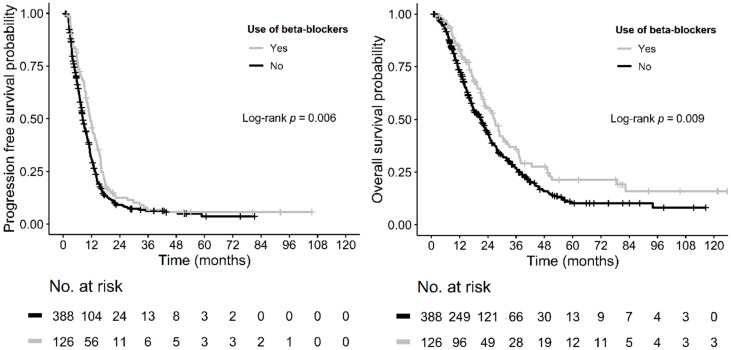
Kaplan-Meier estimates of progression-free survival (PFS) and overall survival (OS) according to the incidental use of beta-blockers.

The univariate Cox analysis evaluating the impact of the use of various antihypertensive drugs on patients’ survival revealed that only beta-blockers were significantly associated with PFS and OS (HR = 0.736 (95% CI 0.592–0.915), *p =* 0.006 and HR = 0.714 (95% CI 0.554–0.921), *p =* 0.009, respectively) (Table 2). The median PFS and OS for patients using beta-blockers were 11.40 months (95% CI 10.10–13.61) and 26.8 months (95% CI 22.2–32.2) compared with 8.30 months (95% CI 7.80–9.57) and 21.0 months (95% CI 17.8–23.8) for patients not using beta-blockers (*p =* 0.006 and *p =* 0.009, respectively) (Table 3, Figure 1).

In the Cox multivariate analysis, the use of beta-blockers was a significant factor predicting both PFS (HR = 0.763 (95% CI 0.606–0.960), *p =* 0.021) and OS (HR = 0.730 (95% CI 0.560–0.951), *p =* 0.020) (Table 4).

## 4. Discussion

The results of this retrospective, single-centre study suggest that the incidental use of beta-blockers is associated with superior PFS and OS in patients with mCRC treated with bevacizumab-based therapy. Apart from beta-blockers, there was no association between outcome of patients and incidental use of other commonly used antihypertensive drugs, including ACE inhibitors, sartans and calcium channel blockers. The multivariate Cox proportional hazards model confirmed that the incidental use of beta-blockers was an independent factor for PFS and OS.

Beta-adrenergic signalling mediates stress responses, also called “fight or flight”, induced by the sympathetic nervous system via catecholamine neurotransmitters represented by adrenaline and noradrenaline. Beta-adrenergic receptors comprising three subtypes (beta-1, beta-2 and beta-3) are constitutively expressed on most mammalian cells, including cancer cells [19]. Their activation can regulate a wide spectrum of cancer-related signalling pathways within both beta-adrenergic receptor expressing cancer cells and other beta-adrenergic receptor expressing cells present in the tumour microenvironment, such as vascular cells and macrophages [20]. The molecular background of several biological processes mediating beta-adrenergic influences on both cancer cells and the tumour microenvironment leading to tumour progression and metastatic spread have been identified. These cancer-related processes include promotion of cell proliferation [21,22], evasion of apoptosis [13,23], tissue invasion mediated by matrix metalloproteinases (MMP) [24], cancer cell mobilisation and motility [8,25], expression of proinflammatory cytokines such as interleukin 6 (IL-6) and IL-8 by both cancer and immune cells [26,27,28], recruitment of macrophages into the tumour [12] and angiogenesis [24,29,30].

Multiple mechanisms contributing to tumour progression mediated by beta-adrenergic signalling suggest that beta-blockers may act as a promising auxiliary treatment strategy with pleiotropic effects on cancer cells and the tumour microenvironment. Various anticancer effects of beta-blocker treatment have been previously demonstrated in experimental models of CRC, pancreatic cancer, ovarian cancer, breast cancer and angiosarcoma [9,10,11,12,13,14]. However encouraging the results from experimental studies may be, the role of beta-blockers in cancer patients remains unclear and the results from mostly retrospective observational studies are equivocal. There are studies suggesting the use of beta-blockers may be able to prolong survival of cancer patients [31,32,33,34,35,36,37,38,39,40,41,42,43,44,45,46,47,48,49]. On the other hand, there are also studies that do not support such a hypothesis [50,51,52,53,54,55,56,57,58,59,60]. Similarly, the results from meta-analyses are ambiguous [61,62,63]. Regarding the role of beta-blocker use in patients with CRC, limited data have been obtained from several retrospective studies with inconclusive results. Moreover, scant data are available for patients with mCRC, particularly those treated with targeted agents. Jansen et al. previously reported a stage-specific association between beta-blocker use and prognosis in patients with CRC in a large population-based retrospective study [42]. They did not find any significant effect of beta-blockers on OS, CRC-specific survival and recurrence-free survival in the whole patient population [42]. However, they did report a significantly longer OS (HR: 0.50, *p* = 0.0023) and CRC-specific survival time (HR: 0.47, *p* = 0.0017) for the beta-blocker users in a subgroup of 256 patients with mCRC [42]. On the other hand, two other large retrospective studies did not find such an association [59,60].

The role of beta-blockers in patients with mCRC treated with antiangiogenic targeted agents is unclear. It has been demonstrated that beta-adrenergic signalling can induce tumour angiogenesis by several distinct molecular mechanisms, including upregulation of VEGF expression in a hypoxia-inducible factor 1-alpha (HIF-1-alpha)-dependent manner and also expression of several other proangiogenic factors, such as IL-6, IL-8, MMP-2 and MMP-9 [24,26,27,28,29,30]. Beta-blockers have been shown to result in decreased VEGF expression and, thus, to an inhibition of angiogenesis [24,64,65]. The antiangiogenic effects of beta-blockers were clearly demonstrated on patients with infantile haemangiomas, in whom treatment with propranolol is commonly used with high efficacy [66,67]. These findings support the hypothesis that the use of beta-blockers could act synergistically with antiangiogenic targeted therapies and could improve the outcomes of cancer patients. To the best of our knowledge, until now, there has been only one retrospective study investigating the role of beta-blocker use in mCRC patients treated with bevacizumab [43]. The study, conducted by Giampieri et al., included 235 mCRC patients treated with chemotherapy alone or with bevacizumab and showed significantly longer OS (HR: 2.26, *p* = 0.003) and a higher response rate (*p* = 0.044) for patients using beta-blockers in the subgroup treated with chemotherapy, while no significant differences were seen in the subgroup treated with bevacizumab [43]. The results of our study contrast with those reported by Giampieri et al. However, the limited number of patients included in their study should be pointed out, in particular those treated with bevacizumab. That study included only 107 patients treated with bevacizumab and only 9 of them used beta-blockers [43]. Thus, the results could be affected by heterogeneity and the small size of the cohort. The strengths of our study are the use of a relatively large cohort of patients and that the patients were diagnosed and treated with a similar strategy and under similar conditions at a single institution.

The present study has several limitations, including its retrospective design, the heterogeneity of the chemotherapy backbone regimens and the dosage and length of beta-blocker exposure not being assessed. Another limitation is that the addition of beta-blockers after initiation of bevacizumab-based therapy was not assessed. Therefore, the patients to whom a beta-blocker was given during the course of bevacizumab-based therapy were not included in the beta-blocker user cohort. This could affect the results because it has been reported that new manifestation or worsening of pre-existing arterial hypertension induced by bevacizumab is associated with a favourable outcome in mCRC patients [68]. On the other hand, the inclusion of these patients might introduce immortal time bias. Our study did not include a cohort treated with chemotherapy alone, and, therefore, it cannot be concluded with certainty that the use of beta-blockers augments the efficacy of bevacizumab or whether it could also improve prognosis of patients treated with chemotherapy alone or chemotherapy combined with other targeted agents. This question should be answered in prospective studies in the future. Nevertheless, this is the largest study published so far evaluating the association between incidental use of beta-blockers and outcome of mCRC patients treated with bevacizumab-based therapy.

## 5. Conclusions

The results of the present retrospective study suggest that there is a significant association between the use of beta-blockers and favourable outcome of mCRC patients treated with bevacizumab-based therapy. The efficacy and safety of the combination of beta-blockers with bevacizumab-based therapy should be investigated in a prospective controlled clinical trial in the future. Our study also indirectly indicates that beta-blockers might be a preferred type of antihypertensive drug for patients with mCRC, especially in those considered for treatment with bevacizumab.

## Figures and Tables

**Table 1:** Baseline patient characteristics.

Characteristic	Category	Use of Beta-Blockers		Overall
		No	Yes	
**Gender**				
	Male	248 (63.9%)	90 (71.4%)	338 (65.8%)
	Female	140 (36.1%)	36 (28.6%)	176 (34.2%)
**Age**				
	Median (range)	62.3 (28.0, 86.1)	65.8 (39.9, 82.6)	63.2 (28.0, 86.1)
	<70 years	309 (79.6%)	98 (77.8%)	407 (79.2%)
	≥70 years	79 (20.4%)	28 (22.2%)	107 (20.8%)
**ECOG PS**				
	0	39 (10.1%)	9 (7.1%)	48 (9.3%)
	1	346 (89.2%)	116 (92.1%)	462 (89.9%)
	2	3 (0.8%)	1 (0.8%)	4 (0.8%)
**Primary tumour location**				
	Left	285 (73.5%)	90 (71.4%)	375 (73.0%)
	Right	88 (22.7%)	32 (25.4%)	120 (23.3%)
	Transversum	15 (3.9%)	4 (3.2%)	19 (3.7%)
**Line of therapy**				
	First line	329 (84.8%)	114 (90.5%)	443 (86.2%)
	Second line	56 (14.4%)	12 (9.5%)	68 (13.2%)
	Third line	3 (0.8%)	0 (0%)	3 (0.6%)
**Chemotherapy**				
	FOLFIRI or XELIRI	72 (18.6%)	19 (15.1%)	91 (17.7%)
	FOLFOX or XELOX	206 (53.1%)	74 (58.7%)	280 (54.5%)
	Other	69 (17.8%)	24 (19.0%)	93 (18.1%)
	Data not available	41 (10.6%)	9 (7.1%)	50 (9.7%)
**Lines of subsequent chemotherapy**				
	No	228 (58.8%)	69 (54.8%)	297 (57.8%)
	One	102 (26.3%)	35 (27.8%)	137 (26.7%)
	Two	39 (10.1%)	17 (13.5%)	56 (10.9%)
	Three	16 (4.1%)	5 (4.0%)	21 (4.1%)
	Four	3 (0.8%)	0 (0%)	3 (0.6%)
**Lines of subsequent chemotherapy combined with targeted therapy**				
	No	302 (77.8%)	100 (79.4%)	402 (78.2%)
	One	85 (21.9%)	23 (18.3%)	108 (21.0%)
	Two	1 (0.3%)	3 (2.4%)	4 (0.8%)
**Lines of subsequent targeted therapy**				
	No	291 (75%)	98 (77.8%)	389 (75.7%)
	One	81 (20.9%)	26 (20.6%)	107 (20.8%)
	Two	15 (3.9%)	1 (0.8%)	16 (3.1%)
	Three	1 (0.3%)	1 (0.8%)	2 (0.4%)
**Synchronous metastases**				
	No	160 (41.2%)	58 (46.0%)	218 (42.4%)
	Yes	228 (58.8%)	68 (54.0%)	296 (57.8%)
**Arterial hypertension**				
	No	215 (55.4%)	99 (78.6%)	272 (52.9%)
	Yes	173 (44.6%)	27 (21.4%)	242 (47.1%)
**Ischaemic heart disease**				
	No	369 (95.1%)	96 (76.2%)	465 (90.5%)
	Yes	19 (4.9%)	30 (23.8%)	49 (9.5%)
**Diabetes mellitus**				
	No	327 (84.3%)	88 (69.8%)	415 (80.7%)
	Yes	61 (15.7%)	38 (30.2%)	99 (19.3%)
**Chronic obstructive pulmonary disease**				
	No	383 (98.7%)	122 (96.8%)	505 (98.2%)
	Yes	5 (1.3%)	4 (3.2%)	9 (1.8%)
**Cancer duplicity**				
	No	365 (94.1%)	104 (82.5%)	469 (91.2%)
	Yes	23 (5.9%)	22 (17.5%)	45 (8.8%)
**Chronic renal failure**				
	No	381 (98.2%)	122 (96.8%)	503 (97.9%)
	Yes	7 (1.8%)	4 (3.2%)	11 (2.1%)
Antihypertensive medication *				
	Beta-blockers	126 (24.5%)		
	Angiotensin-converting-enzyme inhibitors	163 (31.7%)		
	Angiotensin II receptor blockers	48 (9.3%)		
	Calcium channel blockers	102 (19.8%)		

* Patients with more antihypertensive medications were included in all relevant subgroups. ECOG PS: Eastern Cooperative Oncology Group performance status.

**Table 2:** Univariate Cox proportional hazards model assessing the impact of antihypertensive medication on progression-free survival and overall survival.

Medication	Progression-Free Survival (PFS)		Overall Survival (OS)	
	HR (95% CI)	*p*-Value	HR (95% CI)	*p*-Value
Angiotensin-converting-enzyme inhibitors	0.932 (0.763–1.139)	0.491	0.841 (0.669–1.056)	0.136
Beta-blockers	0.736 (0.592–0.915)	0.006	0.714 (0.554–0.921)	0.009
Angiotensin II receptor blockers	0.847 (0.610–1.178)	0.324	1.049 (0.739–1.488)	0.789
Calcium channel blockers	0.886 (0.700–1.121)	0.313	0.932 (0.716–1.214)	0.603

HR: hazard ratio; CI: confidence interval.

**Table 3:** Progression-free and overall survival according to the use of beta-blockers.

Survival	Use of Beta-Blockers		*p*-Value
	No	Yes	
Median PFS (95% CI)	8.30 months (7.80–9.57)	11.40 months (10.10–13.61)	0.006
3-month PFS (95% CI)	0.894 (0.863–0.925)	0.960 (0.926–0.995)	
6-month PFS (95% CI)	0.665 (0.619–0.714)	0.766 (0.695–0.844)	
12-month PFS (95% CI)	0.309 (0.264–0.361)	0.472 (0.392–0.571)	
18-month PFS (95% CI)	0.136 (0.104–178)	0.185 (0.126–0.272)	
Median OS (95% CI)	21.00 months (17.8–23.8)	26.8 months (22.20–32.20)	0.009
12-month OS (95% CI)	0.734 (0.689–0.781)	0.825 (0.760–0.896)	
24-month OS (95% CI)	0.439 (0.388–0.497)	0.553 (0.465–0.656)	
36-month OS (95% CI)	0.269 (0.223–0.326)	0.357 (0.271–0.471)	

**Table 4:** Multivariate Cox proportional hazards model for progression-free and overall survival.

Parameter	Category	Progression-Free Survival (PFS)		Overall Survival (OS)	
		HR (95% CI)	*p*-Value	HR (95% CI)	*p*-Value
Gender	Male	1.000	0.985	1.000	0.302
	Female	0.998 (0.805–1.238)		1.133 (0.894–1.437)	
Age	<70 years	1.000	0.459	1.000	0.813
	≥70 years	1.100 (0.855–1.414)		1.034 (0.781–1.370)	
ECOG PS	0	1.000		1.000	
	1	1.066 (0.776–1.464)	0.693	1.136 (0.782–1.650)	0.503
	2	0.614 (0.183–2.058)	0.430	0.828 (0.244–2.810)	0.763
Primary tumour location	Left	1.000	0.239	1.000	0.361
	Right	1.152 (0.910–1.460)		1.131 (0.868–1.476)	
Line of therapy	First line	1.000		1.000	
	Second line	1.127 (0.816–1.557)	0.468	1.011 (0.706–1.447)	0.954
	Third line	3.021 (0.956–9.551)	0.060	2.199 (0.694–6.974)	0.181
Chemotherapy	FOLFIRI or XELIRI	1.000		1.000	
	FOLFOX or XELOX	0.782 (0.693–1.013)	0.062	0.656 (0.496–0.870)	0.003
	Other	0.788 (0.563–1.101)	0.163	0.854 (0.602–1.211)	0.375
Synchronous metastases	No	1.000	0.500	1.000	0.743
	Yes	0.932 (0.759–1.144)		0.963 (0.768–1.207)	
Use of beta-blockers	No	1.000	0.021	1.000	0.020
	Yes	0.763 (0.606–0.960)		0.730 (0.560–0.951)	

ECOG PS: Eastern Cooperative Oncology Group performance status.
